# Facilitators and barriers to effective primary health care in Nigeria

**DOI:** 10.4102/phcfm.v10i1.1641

**Published:** 2018-02-21

**Authors:** Abraham N. Gyuse, Agam E. Ayuk, McSteve C. Okeke

**Affiliations:** 1 Department of Family Medicine, University of Calabar, Nigeria; 2 Department of Family Medicine, University of Calabar Teaching Hospital, Nigeria

## Demographics

Nigeria is the most populous African nation with an estimated population of 182 million citizens in 2016.^1^ The population distribution is mostly rural (and agrarian), although there are large cities like Lagos, Ibadan, Kano, Port Harcourt and Abuja. The country has over 374 ethnic groups with Hausa, Yoruba, Ibo, Nupe and Tiv being the leading five languages. The official unemployment rate is 14% in the last quarter of 2016^2^; however, the youth unemployment rate was given by the National Bureau of Statistic to be 47% in the same period. This implies that the real unemployment rates are much higher than given by the government for political expediency. The life expectancy is 53 years for men and 56 years for women.

Nigeria is traditionally agrarian, with the agricultural sector accounting for almost 20% of the total gross domestic product (GDP) in 2013. However, since 1980 oil production has accounted for more than two-thirds of GDP and more than 80% of total government revenue. The country is the 12th largest producer of petroleum in the world, is the 8th largest exporter, has the 10th largest proven reserves and is also a founding member of the Organization of Petroleum Exporting Countries.^3,4^

Nigeria is made up of 36 states, 774 local government areas (LGAs) and a Federal Capital Territory in Abuja, the seat of government. The country operates a federal system of government with a bicameral National Assembly.

## Structure of primary health care

All three tiers of government (federal, state and local) share responsibilities for providing health services in Nigeria. The federal government is largely responsible for providing policy, planning and technical assistance, coordinating state-level implementation of the National Health Policy and establishing health management information systems. In addition, the federal government is responsible for disease surveillance, drug regulation, vaccine management and training health professionals. It is also responsible for the management of teaching, psychiatric and orthopaedic hospitals and some medical centres.

The responsibility for the management of health facilities and programmes is shared by the State Ministries of Health, State Hospital Management Boards and the LGAs. The states operate the secondary health facilities (general hospitals) and, in some cases, tertiary hospitals, as well as some primary health care facilities. The training of nurses, midwives and health technicians and the provision of technical assistance to local government health programmes and facilities are also the responsibility of the state authorities.

The local governments oversee the operations of primary health care facilities within their geographic areas. This includes the provision of basic health services, community health, hygiene and sanitation.

The inadequacy of the public health system has given increasing prominence to the private health sector as well as to traditional and spiritual healers. The private sector includes for-profit services, faith-based non-profit services and corporate-based services.

Primary care in Nigeria is delivered and accessed through primary, secondary and tertiary health facilities, while in rural areas primary care is mostly situated in governmental primary health care centres and faith-based clinics. The location of primary care in referral hospitals is mainly because of the shortage of health care providers. Physicians and other health care providers work with whatever health staff is available to offer primary care to the population where they are located. Specialists may also practise primary care in private practices. Although this scenario has afforded the citizens some measure of health care, the system is chaotic and does not allow for quality services.

Primary health care (PHC) services are adapted and modified to suit local needs and cultures. Consequently, churches and mosques, traditional birth attendants (TBAs), village health workers, community leaders, prominent citizens and civil society organisations and groups may all get involved in the delivery of primary health care.

The country has made several attempts at implementing the Alma Ata declaration with different strategies from 1975 to date. PHC has gone through various phases of fragmented implementation involving a multitude of different organisational structures at different levels of the health system. The 54th National Council of Health meeting in May 2011 approved the policy to bring Primary Health Care Under One Roof (PHCUOR).^5^ This policy would integrate all services under one authority in accordance with the World Health Organization guidelines for integrated district-based service delivery.^6^

The *National Health Act 2014* is the first in the history of the country and continues to maintain the basic focus of the national health policy on PHC as central to providing health for all. The Act creates a basic health care provision fund (not less than 1% of federal government consolidated revenue fund). Fifty per cent of this fund will be disbursed by a National Health Insurance Scheme (NHIS) to provide a basic minimum package of health services to citizens. The remaining 50% will provide essential drugs, vaccines and consumables, and infrastructure; develop human resources; and ensure emergency medical treatment at the PHC level.^7^ This initiative has also compelled all levels of health care to revitalise the concept of a basic health unit, made of a community health centre and four clinics to serve 150 000 people. Currently, the federal government is revitalising and committed to building 10 000 new primary care facilities across Nigeria.

## Disciplines working in primary health care in the community

**FIGURE 1:**
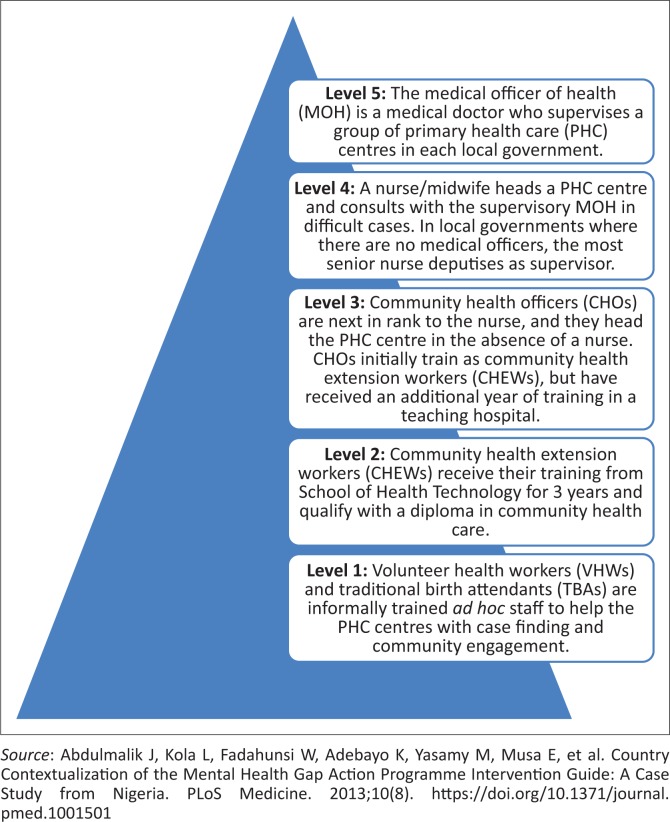
Organisational structure of the Nigerian primary health care system.

There are several health disciplines working in the community to provide health services. These include family physicians (FP), public health physicians, general practitioners (GP), nurses and midwives, pharmacy or pharmacy technicians, laboratory technicians, community health extension workers (CHEWs) and voluntary health workers. A variety of medical specialists, traditional (birth attendants and bone setters), alternative (patent medicine vendors) and allied health professionals (physiotherapists and social workers) may also be present. In general, there is no restriction on who can participate in PHC. The official structure of the PHC system is shown in Figure 1. The distribution of health workers is skewed in favour of the southern part of the country and urban centres.

## Impact on health outcomes

Health indicators in Nigeria have remained below country targets and international benchmarks including the Millennium Development Goals.^8^ Disability-adjusted life expectancy at birth is 39 years. Vaccine-preventable and infectious diseases are still rampant. Nigeria has the fourth highest tuberculosis burden in the world. Non-communicable diseases and neglected tropical diseases are on the increase. Under-five mortality is still 128 per 1000 live births. Only 25% of children are fully immunised (as at 1 year of age). Only 36% deliver in a health facility. Maternal deaths contribute 32% of death among women aged 15–49 years.^9^ It is obvious given the massive oil wealth and resources injected into the health system over many years that the outcomes are not commensurate with the inputs.

## Training the primary care workforce

Almost all medical schools are incorporating PHC into the curriculum as required by the National University Commission (NUC). There is an increasing number of medical schools creating academic departments of family medicine to cater for this need. Enrolment into the residency training programmes in family medicine has been on the increase with several new programmes opened across the country. Family medicine as a specialty has been recognised by the Nigerian government through the activities of the Society of Family Physicians of Nigeria (SOFPON) as an important partner in PHC delivery as FP should be able to provide clinical leadership to the PHC team.

Since the early 1980s, government at all levels has invested in the training of the PHC workforce through the Community Health Officers’ (CHOs) training programme in most teaching hospitals across the country. There are also Colleges of Health Technology to train other PHC workers, schools of nursing and midwifery in all states and the Federal Capital Territory to train the needed nurses and midwives.

## What barriers are encountered?

The country has several barriers that limit delivery of PHC: internal conflict in parts of the country, crime and corruption, multiplicity of governmental and donor agencies, vertical programmes, low political commitment to implementation of approved health policies, differences in remuneration between levels of care, inequality in infrastructure that favours urban areas, poor working conditions, maldistribution of health care workers including emigration, inadequate training facilities in parts of the country and inter-professional conflict.

## Lessons for other countries

Lessons from Nigeria regarding PHC include establishment of *National Health Act* to achieve universal health coverage and health insurance, integration of PHC activities under one authority (PHCUOR), provision of basic health plan and ward health system for rural and grassroots health delivery and the establishment of schools of nursing or midwifery and college of health technologies for training midlevel health workers.
